# What is a biosecurity measure? A definition proposal for animal production and linked processing operations

**DOI:** 10.1016/j.onehlt.2022.100433

**Published:** 2022-09-16

**Authors:** Nikolaus Huber, Mathieu Andraud, Elena L. Sassu, Christopher Prigge, Veit Zoche-Golob, Annemarie Käsbohrer, Daniela D'Angelantonio, Arvo Viltrop, Jacek Żmudzki, Hannah Jones, Richard P. Smith, Tijs Tobias, Elke Burow

**Affiliations:** a Farm Animals and Veterinary Public Health, University of Veterinary Medicine (Vetmeduni), Vienna, Austria; b Ploufragan-Plouzané Laboratory, Agence nationale de sécurité sanitaire de l'alimentation, de l'environnement et du travail (ANSES), France; c Division for Animal Health, Austrian Agency for Health and Food Safety (AGES), Austria; d Department of Biological Safety, German Federal Institute for Risk Assessment (BfR), Berlin, Germany; e Istituto Zooprofilattico Sperimentale dell'Abruzzo e del Molise G. Caporale (IZSAM), Italy; f Institute of Veterinary Medicine and Animal Sciences, Estonian University of Life Sciences, Estonia; g Department of Swine Diseases, National Veterinary Research Institute, Poland; h Animal and Plant Health Agency, United Kingdom; i Department Population Health Sciences, Faculty of Veterinary Medicine, Utrecht University, Utrecht, Netherlands

**Keywords:** Biosecurity, One health, Livestock, Swine, Zoonotic disease prevention

## Abstract

While biosecurity, a central component of the One Health concept, is clearly defined, a harmonized definition of the term ´biosecurity measure´ (BSM) is missing. In turn, particularly at the farm and policy level, this leads to misunderstandings, low acceptance, poor implementation, and thus suboptimal biosecurity along the food animal production chain. Moreover, different views on BSMs affects making comparisons both at the policy level as well as in the scientific community. Therefore, as part of the One Health EJP BIOPIGEE project, a work group i) collected and discussed relevant inclusion and exclusion criteria for measures to be considered in the context of biosecurity and ii) conducted a systematic literature review for potentially existing definitions for the term BSM. This exercise confirmed the lack of a definition of BSM, underlining the importance of the topic. In the pool of articles considered relevant to defining the term BSM, specific research themes were identified. Based on these outcomes, we propose a definition of the term BSM:

“A biosecurity measure (BSM) – is the implementation of a segregation, hygiene, or management procedure (excluding medically effective feed additives and preventive/curative treatment of animals) that specifically aims at reducing the probability of the introduction, establishment, survival, or spread of any potential pathogen to, within, or from a farm, operation or geographical area.”

The definition provides a basis for policymakers to identify factual BSMs, highlights the point of implementation and supports to achieve the necessary quality standards of biosecurity in food animal production. It also enables clear, harmonized, cross-sectoral communication of best biosecurity practices to and from relevant stakeholders and thus contribute to improving biosecurity and thereby strengthen the One Health approach.

## Highlights

• We propose a harmonized definition for the term “biosecurity measure”.
• Policy makers can use this definition to identify factual BSMs.
• The definition promotes communication of biosecurity practices between stakeholders.

## 1 Introduction

The global increase in demand for animal protein entails more geographically concentrated and intensive animal production. The high density of animal production sites and their related contact structures are drivers for infectious animal diseases responsible for morbidity, mortality, and economic losses worldwide [[1], [2], [3]]. Biosecurity is one of the essential components of the One Health concept [4,5]. It is considered critical in this context, as it aims to prevent infections and their spread to farmed animals, humans, and the environment (including wildlife and plant species) and, by safeguarding health and well-being, to curtail the impact of infectious diseases on the environment, the economy, and society in general. Additionally, the increase in movement of live animals and animal products, as well as the diversification and expansion of food supply chains, represents a risk for public health due to the possible emergence and spread of foodborne and zoonotic agents [[6], [7], [8]]. Mitigating these risks necessitates strategies towards improved husbandry and health management to i) prevent zoonotic disease outbreaks in animals and humans and ii) ensure food safety and secure public health [9]. However, the current SARS-CoV-2 pandemic, as the most recent prominent example, and the emergence and re-emergence of infectious diseases such as salmonellosis, avian influenza, or African swine fever demonstrate that the development and successful implementation of biosecurity strategies are complex and continuous challenges [3,10].

The concept of biosecurity per se has been included across various sectors and scales, from the environmental to the animal and human health sector, and from the individual animal/human to farm/operation, regional, national, and international scale. Biosecurity is a part of several strategic documents in animal and public health and policy for livestock production [11]. In the European Animal Health Law (AHL; Regulation (EU) 2016/429) biosecurity is defined as: “the sum of management and physical *measures* designed to reduce the risk of the introduction, development and spread of diseases to, from and within: (a) an animal population, or (b) an establishment, zone, compartment, means of transport or any other facilities, premises or location” [12]. The Food and Agriculture Organization of the United Nations (FAO) defines biosecurity as the”implementation of *measures* that reduce the risk of the introduction and spread of disease agents; it requires the adoption of a set of attitudes and behaviours by people to reduce risk in all activities involving domestic, captive/exotic and wild animals and their products” [13]. “At the farm level, *biosecurity measures* may focus either on reducing the risk of entry of new pathogens (external biosecurity) or on reducing the internal dissemination of pathogens (internal biosecurity)” [13]. The World Organization for Animal Health (OIE) defines biosecurity in the Terrestrial Animal Health code as “a set of management and physical *measures* designed to reduce the risk of introduction, establishment and spread of animal diseases, infections or infestations to, from and within an animal population” [14].

As part of the One Health European Joint Programme (OHEJP), the project “Biosecurity practices for pig farming across Europe” (BIOPIGEE) aims to establish a best practice protocol of effective biosecurity measures (BSMs) to reduce the occurrence of two critical infectious and zoonotic pathogens, *Salmonella* spp. and hepatitis E virus (HEV), in the pig production chain. To achieve this aim, 12 European countries and 19 research institutions, including several scientific disciplines (i.e., epidemiology, microbiology, bacteriology, veterinary and human medicine, as well as agronomy and econometrics), collaborated. However, the categorization and evaluation of the various measures referred to as BSMs for the occurrence of *Salmonella* and HEV in pig production were often unclear. Therefore, determining whether the indicated measures contribute to the prevention of *Salmonella* and HEV in pork production systems was not always possible.

While it is defined clearly what “biosecurity” is, a harmonized definition for BSM is lacking. Consequently, the question “What is a BSM, exactly?” arises. In this context, Kuster et al. [15] point out a “need for more precise and commonly accepted definitions of biosecurity measures.” The lack of a standard definition of BSM and what measures can be considered BSMs affect the understanding, acceptance, and broad, effective implementation and comparable evaluation of effectiveness of these measures at different levels of the food production chain. [[16], [17], [18]]. Therefore, a clear and precise definition of BSMs is essential to achieve the necessary quality standards of biosecurity in pig/animal and food production to protect and improve human and animal health and consequently the wider environment in a sustainable and reliable way. Moreover, a clear and concise definition of BSM is necessary to improve communication of the best biosecurity practices to and from relevant stakeholders. Schlundt et al., concluded that “Future achievements in food safety, public health and welfare will largely be based on how well politicians, researchers, industry, national agencies and other stakeholders manage to collaborate using the One Health approach” [19]. A key prerequisite for achieving effective results and defined goals within these essential collaborations and One Health networks is the clear and unambiguous communicability of the necessary tools such as BSMs.

This manuscript aims to provide a standard definition for the term “BSM” in the context of animal production systems and related processing operations that can contribute to a common understanding and better communication between sectors within the One Health domain.

## 2 Methodology

We (i) collected and discussed potentially relevant biosecurity-related inclusion and exclusion criteria for BSM. In parallel, we (ii) performed a systematic literature search using a scoping review approach for potentially existing definitions of BSM in the pig sector. Thereafter (iii), results of (i) and (ii) were used in an iterative process to propose a definition of BSM.

### 2.1 Collection and discussion of in- and exclusion criteria

A working group with representatives of epidemiology, microbiology, bacteriology, veterinary medicine biology, and agronomics (10 experts) collected the criteria applied to include or exclude activities related to biosecurity in their respective task groups (Table 1). The collected criteria were discussed in several online video meetings and evaluated for relevance towards a definition of the BSM, whereby organizational or study population criteria, such as “commercial pig farms only,” were not considered. The remaining criteria were summarized, and along with a preliminary definition chart containing determining and influencing factors linked to the key fragments of the definition, a first working definition of the term BSM was proposed for the iterative process.

**Table 1:** Overview and description of work groups within the One Health European Joint Programme project BIOPIGEE. All of the working groups outlined address Salmonella and Hepatitis E Virus (HEV) control, and were used to collect the inclusion and exclusion criteria relevant to the definition of the term biosecurity measure.

Work group	Task Description
Biosecurity effectiveness studies	
WP2.1	Development of a biosecurity protocol for pig farms
WP2.2	Application of the biosecurity protocol on pig farms
WP2.3	Slaughterhouse biosecurity practices
Benchmark of biosecurity practice	
WP5.2	Systematic literature review and meta-analysis of biosecurity measures in pig farms (additionally addressing pathogenic *E. coli*)
WP5.4	Expert panel to add estimations on effectiveness/weights of biosecurity practices to BSM catalogue of the project

### 2.2 Literature scoping review

**Fig. 1:**
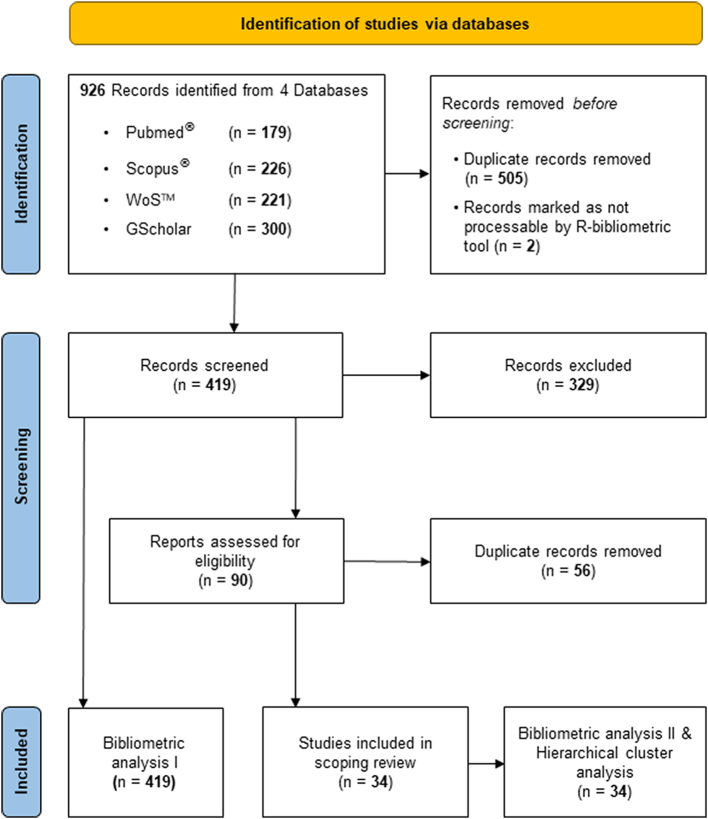
PRISMA flow diagram for literature search progression applying the search terms “biosecurity measures” AND “pig OR swine”, adopted from [53]. After duplicates were removed, 419 articles remained, of which a total of 90 articles were manually extracted (one researcher per database) because they contained relevant information for the definition of biosecurity measure (329 were not considered further and excluded). In the next step, articles that were manually selected in duplicate by independent researchers (e.g., in both Pubmed and Scopus) were cleared of these duplicates, resulting in 34 final articles.

Another working group with representatives of epidemiology, biology, microbiology and veterinary medicine (4 experts) conducted a systematic literature scoping review. The search terms “biosecurity measures” AND “pig OR swine” in titles, abstracts, or keywords were applied in the Scopus, PubMed®, Web of Science™, and Google Scholar (GS) databases by one independent researcher each (access dates: 15th of May 2021). Because the GS search algorithm does not specify term location resulting in >5000 articles, the first 300 articles sorted by relevance were selected to achieve a comparable number of article hits as in the other databases (Fig. 1). After removing duplicates between databases, the remaining articles were processed to extract bibliometric information, such as year and type of publication, open access vs. non-open access, authoring, key wording, and networking [20]. In a second step of the review process, each database was processed by a single researcher with the objective of extracting up to 25 articles from each database that specifically addressed BSMs in swine production systems or included a description comparable to or close to a definition of the term. Each researcher conducted the selection independently to ensure that the process was not biased.

A second bibliometric analysis was performed on the final subset of records to assess the specificity of our selection process towards the term of interest when compared to the initial raw record set. Full-text articles were then extracted and prepared for R-based text mining tools by removing punctuation, numbers, and stop words as well as converting all text to lowercase. A document-term matrix (DTM) was built with all words appearing on average more than three times in the corpus, or with term occurrence higher than 100 times. A hierarchical cluster analysis was performed on this DTM to identify common traits regarding scientific context and objectives among the selected articles. Based on the descriptions of BSMs in the final records and the above-mentioned analyses, the group proposed a working definition for the term BSM.

### 2.3 Expert panel/definition working group

In an iterative process, the developed flowchart and the working definitions proposed by the two working groups were discussed by all participants of both working groups in several joint meetings. This was completed to reach one harmonized definition of BSM and refinement of the flowchart showing the individual definition components as well as the determining and the potential influencing factors associated with these components. Next, the result was presented and discussed in an expert workshop (OHEJP workshop, December 2021; 62 participants from 11 European countries including 17 members of the OHEJP Expert Panel). The input from the participating international experts on biosecurity in different animal production systems was incorporated in the conceptualization and final formulation of the definition for the term BSM.

## 3 Results

### 3.1 Collection and discussion of in- and exclusion criteria

The relevant inclusion and exclusion criteria selected across the OHEJP BIOPIGEE work groups (depicted in Table 1) are summarized in Table 2. In this context, “primary biosecurity” was considered as the prevention of pathogen spread between farms, “secondary biosecurity” as the prevention of pathogen spread within a farm, and “tertiary biosecurity” as measures that increase the resistance (e.g. antimicrobials) or the immunity of the animals against pathogens (e.g., vaccination). Here we would like to note that the terms primary, secondary and tertiary do not refer to the importance or effectiveness of the respective measures in the context of animal or human health.

**Table 2:** Selection of inclusion and exclusion criteria relevant to a definition of the term biosecurity measure (BSM), collected across the One Health European Joint Programme BIOPIGEE project.

Inclusion criteria	Exclusion criteria
• Association to pathogen occurrence in pig operations	▪ Factors requiring major changes of the operation (location of the buildings, size)
• Application of procedures (goal/, pre/post conditions, timing, physical requirements, rules) or physical processes	▪ Procedures for the monitoring of the compliance to, or the effectiveness of BSMs
• Primary and secondary biosecurity^1^, ^2^	▪ Proficiency of personnel
	▪ Description of pathogen status
	▪ Tertiary biosecurity^3^

1 Primary biosecurity: the prevention of pathogen spread between farms.

2 Secondary biosecurity: prevention of pathogen spread within a farm.

3 Tertiary biosecurity: measures that increase the resistance or immunity of the animals against pathogens.

### 3.2 Literature scoping review

**Fig. 2:**
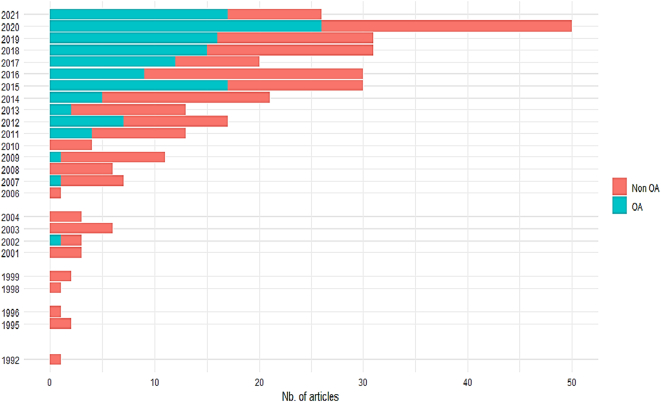
Number of scientific papers (*n* = 419, search terms: “biosecurity measures” AND “pig OR swine”) published from 1992 to May 2021 in either open access (OA, turquoise) or non-open access journals (Non-OA, red), including PubMed®, Scopus, Web of Science (Clarivate Analytics™) and Google scholar scientific literature databases. (For interpretation of the references to colour in this figure legend, the reader is referred to the web version of this article.)

**Fig. 3:**
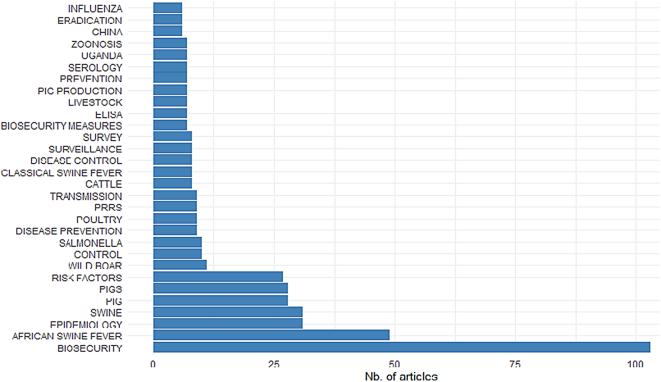
Top Keywords of scientific papers representing the first selection in this study (n = 419; search terms: “biosecurity measures” AND “pig OR swine” including PubMed®, Scopus, Web of Science™ and Google scholar scientific literature databases accessed in May 2021).

In total, 926 records were identified with a considerable overlap across the four different databases, i.e., 505 articles were duplicates (Fig. 1, Table 3A). Two documents, [21] and a report [22] (the authoring being anonymous), could not be processed due to technical incompatibility with the R-bibliometric tools. The bibliometric analysis of the first paper pool (*n* = 419) revealed a steep increase in publications around the topic of biosecurity in the context of pig production and linked processing operations since 1992 (Fig. 2). Keyword occurrences showed a strong pathogen focus on ASF, *Salmonella,* and classical swine fever (CSF) in the context of biosecurity in pig production, but also wild boar, poultry, and cattle are listed as dominant keywords. However, BSM (our term of interest) ranks relatively low compared to the previously mentioned and general terms in the context of disease prevention and more general terms like epidemiology and risk factors (Fig. 3). Moreover, in the second step of the scoping review, a total of 90 articles were finally selected in the four databases, with a large overlap since only 34 publications remained when duplicates were removed (Table 3B; Fig. 1; citations of articles included in the final selection are provided as supplementary material).

**Table 3:**
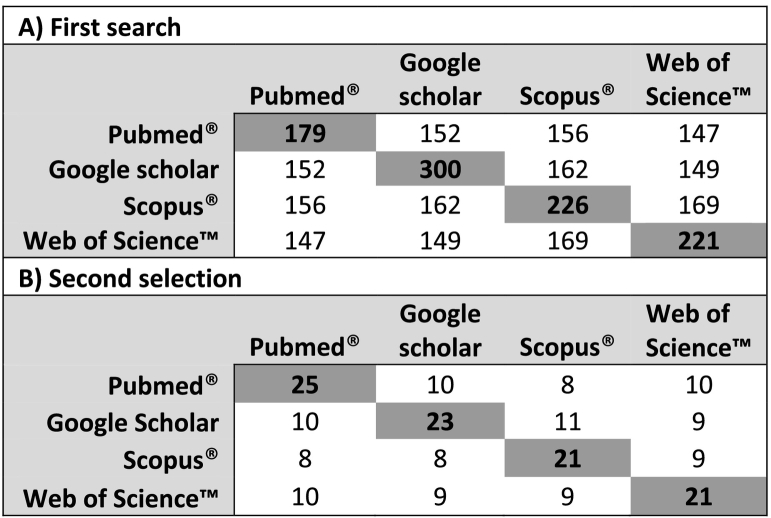
A: Identified records in four scientific literature databases (first search, results (bold and gray shaded numbers, *n* = 926) and overlap of identical records between databases (non-bold, non-shaded numbers) B: Manually and independently selected articles by relevance (bold and gray shaded numbers, *n* = 90) and overlap of selected articles between researchers where one researcher worked one database (non-bold, non-shaded numbers).

**Fig. 4:**
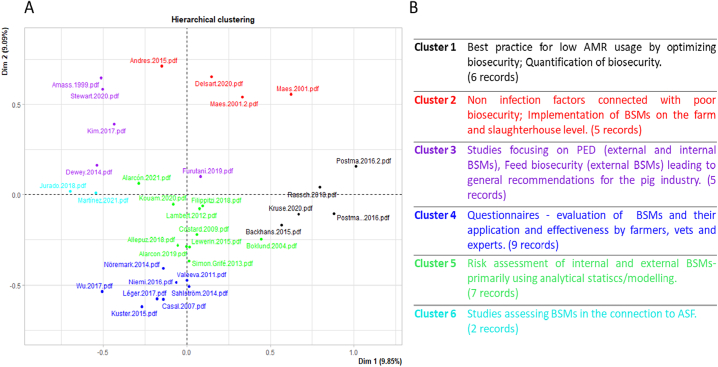
A: Hierarchical cluster analysis based on the occurrence of one of the 100 most frequent terms in an article in the final pool of papers (*n* = 34). B: Description of the six research clusters and the scientific topics they cover revealed by the clustering analysis. After additional full-text reading, the clusters were modified i.e., numbers of records in 4A being not concurrent with the numbers given in 4B.

The hierarchical clustering of the final set of publications resulted in six research clusters, which relate to different scientific contexts (Fig. 4). The first cluster accounted for six records objectivizing the role of BSMs on the reduction of antimicrobial usage on farms. Five records focused on the impact of non-infectious factors on the spread of diseases from farms to slaughterhouses (cluster 2). Environmental conditions favoring pathogen spread and persistence were strongly related to poor sanitation and biosecurity implementation. Another group of five studies was dedicated to BSMs in regard to animal feed and provided recommendations for the pig industry (cluster 3). Nine studies presented a questionnaire-based evaluation of BSMs on farms and their perceived effectiveness by all actors (cluster 4). Risk analysis of BSMs regarding specific infectious agents was performed in seven studies from cluster 5. Finally, cluster 6 accounted for two studies dedicated to ASF (2 studies). These descriptions are based on the interpretation of statistical analysis after full text reads.

Nevertheless, three records were found to be at the crossroads between clusters. Furutani et al., [23] discussed the role of BSMs on the transmission of Porcine epidemic diarrhea virus (PEDV). Although animal feed represents a non-negligible introduction pathway for PEDV the risk for PEDV on different BSMs was assessed and was therefore moved from cluster 3 to cluster 5 (Fig. 4B). The study by Kim et al. [24], focusing on indirect transmission via farm personnel, was moved from cluster 3 to cluster 2. The study by Lewerin et al., [25], which proposed a toolset for assessment of biosecurity on farms, was found to better fit to cluster 4 instead of cluster 5, i.e., the numbers of records in Fig. 4 are not concurrent with the numbers in Fig. 4B [25]. Overall and most importantly, although BSMs appear in a large spectrum of scientific interests, the scoping review did not reveal a clear and usable definition of BSM in the scientific literature.

### 3.3 Expert panel/definition task group

**Fig. 5:**
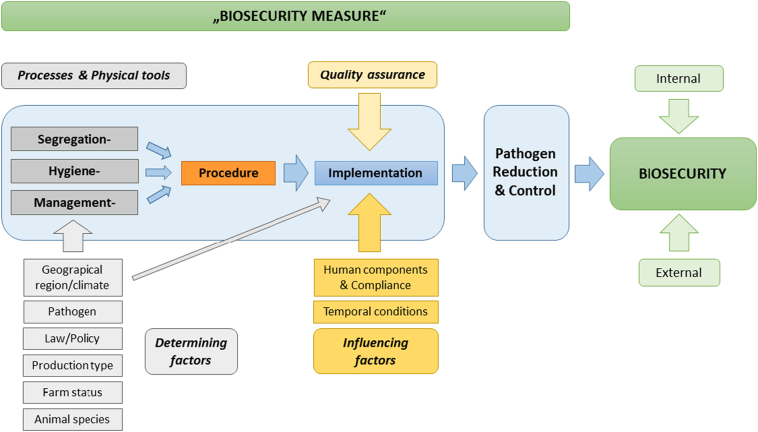
Defintion chart of the term “biosecurity measure” (dark green box) with the overall goal of reducing the probability of pathogen establishment, survival, or spread, thereby contributing to internal or external (light green boxes) biosecurity in animal production systems and related processing operations. Embedded in the two light blue boxes are the key fragments of the proposed definition of BSM. The light gray boxes below the central field, such as “production type” or “animal species” indicate the determining factors for the processes and physical tools (dark gray boxes) that include segregation, sanitation, or management procedures (orange box). In addition, the geographic region and the given climate are determining factors for the respective procedures and influence their implementation (dark blue box). The implementation of these procedures is further influenced by influencing factors (yellow boxes), such as temporal conditions (long- vs. short-term pathogen reduction effects), as well as human components (e.g., attitude or motivation towards the implementation) with the implementation of procedures, which are essential points to ensure the quality (effectiveness) of the respective measure (sand-colored box). (For interpretation of the references to colour in this figure legend, the reader is referred to the web version of this article.)

The results and the two working definitions from both subtask groups and the input of a biosecurity experts received in the frame of the OHEJP BIOPIGEE workshop were then discussed jointly. In the context of animal production systems and associated processing operations, a refined flowchart for the definition of BSM (shown in its agreed form in Fig. 5) and the following definition for the term “BSM” have been developed:

“A biosecurity measure -.

– is the implementation of a segregation, hygiene, or management procedure (excluding medically effective feed additives and preventive/curative treatment of animals) that specifically aims at reducing the probability of the introduction, establishment, survival, or spread of any potential pathogen to, within, or from a farm, a linked processing operation or a geographical area.”

## 4 Discussion

The awareness of biosecurity and its underlying measures is growing, as is their great relevance for livestock production, shown by the sharp increase in publications mentioning the term BSM in the pig sector by almost a factor of five between 2011 and 2021 [4] (Fig. 2). Given the intensification of animal production with worldwide high-frequency transport of animals and their products, the introduction of a new zoonotic pathogen, or the excessive or uncontrolled spread of endemic pathogens on farms or linked processing operations, may have serious to catastrophic consequences for human, animal and environmental health with high economic losses [1,[6], [7], [8]]. However, the top keyword from the final publications in our scoping review depicts the general term biosecurity as predominant, whereas BSM ranks comparatively low (Fig. 3). The large difference in frequency between the two terms may be because biosecurity is well established, frequently addressed, or promoted as essential to animal production. In comparison, BSMs may not be as specifically and frequently addressed in a clear and standardized manner [15,16]. Especially in the last decade, the term biosecurity has been increasingly used and referenced, however, without clearly defining BSM. Despite the wide range of scientific interests represented by the research clusters, no general definition of BSM could be identified in the scientific literature. Some studies, however, describe what BSMs should strive for, such as: “BSMs are important tools to maintain animal health in pig herds” [26]; “the key concept in biosecurity is to avoid transmission, either between farms or within the farm. Therefore, the applicable measures must result in a reduction of the probability of effective transmission“[27]; or “Typically, BSMs focus on eliminating either the pathogen (e.g., disinfection) or the realization of the route which can transmit the pathogen” [17].

The vast majority of studies in the scientific literature from the pig sector, including the final paper pool of our scoping review, all agree on the overarching goal that BSMs contribute to biosecurity by reducing the risk of pathogen introduction and spread [[27], [28], [29], [30], [31], [32]]. This defining feature is considered one of the three most crucial inclusion criteria selected by the OHEJP BIOPIGEE evaluation group (Table 2). There is also broad agreement on where BSMs should contribute within the livestock/food production chain: i) external biosecurity (reducing the risk of entry of pathogens into a farm or operation) and ii) internal biosecurity external (reducing the internal dissemination of pathogens within a farm or operation) [13]. In BIOPIGEE, these two aspects were considered important inclusion criteria contributing to a general definition of BSM. “Primary biosecurity” was defined as the prevention of pathogen spread between farms, and “secondary biosecurity” as the prevention of pathogen spread within a farm. These characteristics are clearly defined in the European Animal Health Law (AHL; Regulation (EU) 2016/429) and in the Food and Agriculture Organization of the United Nations (FAO) definition of biosecurity at the farm level [12,13]. In this context, we would like to point out that the new AHL emphasizes the importance of biosecurity as an essential obligation and responsibility for all operators, i.e., livestock producers and linked processing operations, transporters but also farmers associations, as well as veterinarians, animal professionals and pet keepers (Article 10, 11 and 12, EU AHL) [12].

We used the definition of biosecurity as defined by the World Organization for Animal Health (OIE) and EU AHL as underlying principle to define BSM, which substitutes reducing the risk of pathogen introduction into, from, or within an animal population for internal or external biosecurity, and additionally considers pathogen spread to the surrounding environment [12,14]. In a bigger One Health context, Renault et al. (2021) state that biosecurity should include the reduction of the probability of spread of pathogens to and between animals, plants, humans, and the environment as the biosecurity definition of the OIE may not be explicit enough in mentioning the links with public and environmental health [4]. Given our primary objective of proposing a standard definition of BSM that encompasses the underlying processes for achieving biosecurity, we aimed to keep the proposed definition as parsimonious as possible to i) provide greater clarity regarding the term and ii) to tie in with existing definitions of biosecurity itself without complicating their communication. Moreover, our wording on this point does not prevent it from being utilized within a broader concept of biosecurity in the context of One Health, as proposed by the World Health Organization (WHO): “biosecurity is a strategic and integrated concept that encompasses the policy and regulatory frameworks including instruments and activities that analyses and manage risk in food safety, public health, animal life and health, and plant life and health including associated environmental risk” [33].

Although biosecurity is often referred to as internal or external relative to the organizational unit or structure at hand, we chose not to distinguish in this aspect for the BSM definition as this division does not contribute to the needed clarity regarding the BSM per se at policy and farm level. In addition, certain BSMs, such as cleaning and disinfection or pest control, can be classified as internal and/or external biosecurity.

“Tertiary biosecurity”, defined as measures that increase animal resistance to potential pathogens (i.e., genetics and the immune system), was discussed and seen as an exclusion criterion in the OHEJP BIOPIGEE (Table 2). We incorporated this exclusion criterion in our definition to explicitly emphasize that medically effective interventions should not be considered as BSMs but fall within the scope of preventive or curative veterinary medicine, as pointed out by [34]. Concerning water or feed biosecurity, it may be challenging to classify, e.g., feed form, formulation, or feed particle size, as a BSM. Indeed, meal and wet feed lead to more acidic conditions in the digestive tract creating a less favorable environment for pathogens, consequently increasing animal resistance to potential pathogens [27,35]. On the other hand, some types of wet feed have an intrinsically low pH decreasing contamination during storage and feeding [36,37]. Thus, we emphasize that any feed- or water-related measure that alters animal resistance to pathogens (internal to the animal, including changes in digestive tract pH) should not be classified as BSM, and that measures that reduce the prevalence of pathogens in water or feed as part of the feed production, transport, storage, or feeding process (external to the animal) can be considered BSMs.

We feel this clear distinction is a pivotal addition to the proposed definition of the term BSM because biosecurity ought to pose the very foundation of any disease control strategy. If BSMs are implemented in a combined and sustainable manner it has been shown that preventive or curative veterinary measures such as medically effective nutritional strategies including bacteriocins increasing animal resistance to pathogens, the use of antimicrobials, non-antimicrobial compounds such as probiotics as well as vaccination can be minimized [38,39]. As an example, and given the significant and interdependent dimensions of antimicrobial resistance in humans, animals, and the environment, the importance of BSMs in the One Health approach is highlighted as they reduce the spread of pathogens and infections while reducing the need for antimicrobial use, thus preserving the much needed efficacy of existing antimicrobials against pathogens. As another prominent example only very few vaccines have proven efficacy to reduce pathogen transmission in a group of animals (e.g. PRRSv in pigs) [40]. In fact, quite often the reduction of transmission is only effective when applying vaccination in combination with additional measures (e.g. restrictions of mixing and movement in the case of Aujezsky's disease). Further, vaccination does not prevent the exposure to and infection with pathogens. Consequently, we would like to point out that while we recognize veterinary medical interventions as effective and vital to an overall livestock and human health strategy, they should not be considered or communicated as BSMs to meet biosecurity requirements as part of the One Health approach.

Two further critical fragments of the proposed definition are the terms “*implementation*” and “*procedure*” (procedure = a series of actions conducted in a particular order and way [41]). Procedure in the frame of our proposed definition of BSM is linked to several factors such as the pathogen and animal species, the legislative framework of the respective region, the production type, the existing grade of biosecurity and pathogen prevalence of a farm/operation, as determining factors shaping the respective segregation, hygiene and management procedures [32,34,42] (Fig. 5). Following the updated FAO definition of biosecurity [13], the term “implementation” has been included in the proposed definition to emphasize the importance of the active application of the fundamental procedures to a BSM as a central component (implementation = putting a decision or plan into effect; execution; [43]). Assessments of pathogen prevalence/status, or the presence of pest management plans on a farm or processing operations, are sometimes considered as BSMs [18]. Pathogen status and prevalence assessments are essential but cannot be considered BSMs per se, as they are merely a prerequisite or part of a procedure as described above. Also, the mere existence of pest management and even biosecurity plans does not imply that they are implemented and maintained or that the BSMs involved are of high quality and effective in reducing pathogens and therefore cannot be considered BSMs.

The design and clarity of separation, hygiene, or management procedures contribute to the quality and effectiveness of the BSM [27]. However, the implementation of procedures is influenced by temporal conditions such as short- vs. long-term effects of pathogen reduction and by human components (Fig. 5). These include compliance with procedures, knowledge, and awareness about disease transmission and biosecurity in general, as well as the necessary skills regarding the respective procedure, which may vary with geographical region and climatic conditions [16,44]. However, the above-mentioned human components are essential for ensuring adequate compliance with procedures and quality in implementing BSMs and are thus crucial to their effectiveness. Despite their importance in this regard, the skills and abilities of the operational staff or implementers themselves cannot be considered BSMs as such (Table 2).

As mentioned before, biosecurity (and thus BSMs) has also been highlighted as an essential factor in reducing antimicrobial resistance (AMR), the spread of AMR genes, and anthelmintic resistance [45,46]. The wording of our proposed definition may be a potential limitation in this context. We chose the formulation “of any potential pathogen” to cover both classical pathogens and all pathogens that can act as such, i.e., opportunistic pathogens (bacteria, viruses, fungi, parasites being pathogenic or nonpathogenic depending on host conditions and environmental context) [47,48]. Thereby our definition only indirectly covers the reduction of the likelihood of AMR genes or anthelmintic resistance by i) direct reduction of pathogens, ii) reduction of antimicrobial/anthelmintic usage and iii) the spread from and within a farm/operation or the environment, but is not explicitly stated. A further limitation could be that collected inclusion and exclusion criteria from OHEJP BIOPIGEE have the background of measures against *Salmonella* and HEV, and possibly other measures that could be relevant for other pathogens were not taken into account. Therefore, not all aspects may have been considered or covered in the proposed definition. Furthermore, this study is based on expertise and scientific literature coming from the pig sector. However, despite these potential limitations we advocate that the proposed definition can be applied to other animal species or livestock sectors.

Based on the identified determining factors (animal species, production type, geographical region and climate etc.) for physiological tools and processes and the influencing factors on their implementation, the structured approach of the proposed definition provides more clarity on the requirements for BSMs and helps to define, communicate and assess them more precisely (Fig. 5). Therefore, the definition helps to further improve quality standards in pig/animal production and its products to protect animals, consumers and their associated environment.

Additionally, the definition of BSM supports standardization by adding clarity to regulations and requirements and facilitating better communication among stakeholders and policymakers. However, the AHL foresees the drafting of implementing acts to lay down minimum requirements necessary for the uniform application of Art.10, which lays down the operator's responsibility for animal health and biosecurity measures (EU AHL; Art.10, par. 6). Specifically, an implementing act that defines which biosecurity measures are appropriate depending on different species of kept animals, type of production and geographical location, is currently not available (EU AHL; Art. 10, par 1, point (b)). A clear definition of what a BSM is can facilitate the process of drafting future implementing acts concerning biosecurity measures. Policymakers can start from our proposed definition to classify the respective BMSs and stress the point of implementation along the animal-human-environmental interface to improve biosecurity levels and safeguard animal, human and environmental health alike and thereby strengthen the One Health approach.

## Funding

This manuscript is part of the European Joint Programme One Health EJP. The BIOPIGEE project is funded from the European Union's Horizon 2020 research and innovation programme under Grant Agreement No 773830.

## Ethics statement

The authors confirm that the ethical policies of the journal, as noted on the journal's author guidelines page, have been adhered to. No ethical approval was required as this article is a combination of a scoping review and the outcome of expert discussions within the OHEJP BIOPIGEE project. The article presents a new definition for the term biosecurity measure but no original research data.

## Declaration of Competing Interest

The authors declare that the research was conducted in the absence of any commercial or financial relationships that could be construed as a potential conflict of interest.

## Data Availability

Data will be made available on request.

## Appendix A Supplementary data

Biosecurity measures in swine production systems or including a description comparable or close to a definition of the term extracted manually by four independent researchers in the frame of a scoping review using the search terms "biosecurity measures" AND "pig OR swine". Image 1
