# Supplementary material for: What is a biosecurity measure? A definition proposal for animal production and linked processing operations
Source: One Health. 2022 Sep 16;15:100433. doi: 10.1016/j.onehlt.2022.100433 (PMC9582555; doi:10.1016/j.onehlt.2022.100433)
Supplement: Supplementary file 1 — Biosecurity measures in swine production systems or including a description comparable or close to a definition of the term extracted manually by four independent researchers in the frame of a scoping review using the search terms "biosecurity measures" AND "pig OR swine". [file mmc1.docx]

# **Supplementary Material**

Final selection of publications specifically addressing biosecurity measures in swine production systems or including a description comparable or close to a definition of the term extracted manually by four independent researchers in the frame of a scoping review using the search terms "biosecurity measures" AND "pig OR swine".

Alarcón, L. V., Allepuz, A., & Mateu, E. (2021). Biosecurity in pig farms: a review. Porcine Health Management, 7(1), 1-15. doi: <https://doi.org/10.1186/s40813-020-00181-z>

Alarcón, L. V., Monterubbianesi, M., Perelman, S., Sanguinetti, H. R., Perfumo, C. J., Mateu, E., & Allepuz, A. (2019). Biosecurity assessment of Argentinian pig farms. Preventive veterinary medicine, 170, 104637. doi: <https://doi.org/10.1016/j.prevetmed.2019.02.012>

Allepuz, A., Martín-Valls, G. E., Casal, J., & Mateu, E. (2018). Development of a risk assessment tool for improving biosecurity on pig farms. *Preventive veterinary medicine*, *153*, 56-63. doi: <https://doi.org/10.1016/j.prevetmed.2018.02.014>

Amass, S. F., & Clark, L. K. (1999). Biosecurity considerations for pork production units. *Journal of Swine Health and Production*, *7*(5), 217-228.

Andres, V. M., & Davies, R. H. (2015). Biosecurity measures to control Salmonella and other infectious agents in pig farms: a review. *Comprehensive Reviews in Food Science and Food Safety*, *14*(4), 317-335. doi: <https://doi.org/10.1111/1541-4337.12137>

Backhans, A., Sjölund, M., Lindberg, A., & Emanuelson, U. (2015). Biosecurity level and health management practices in 60 Swedish farrow-to-finish herds. *Acta Veterinaria Scandinavica*, *57*(1), 1-11. doi: <https://doi.org/10.1186/s13028-015-0103-5>

Boklund, A., Alban, L., Mortensen, S., & Houe, H. (2004). Biosecurity in 116 Danish fattening swineherds: descriptive results and factor analysis. *Preventive Veterinary Medicine*, *66*(1-4), 49-62. doi: <https://doi.org/10.1016/j.prevetmed.2004.08.004>

Casal, J., De Manuel, A., Mateu, E., & Martin, M. (2007). Biosecurity measures on swine farms in Spain: perceptions by farmers and their relationship to current on-farm measures. *Preventive veterinary medicine*, *82*(1-2), 138-150. doi: <https://doi.org/10.1016/j.prevetmed.2007.05.012>

Costard, S., Porphyre, V., Messad, S., Rakotondrahanta, S., Vidon, H., Roger, F., & Pfeiffer, D. U. (2009). Multivariate analysis of management and biosecurity practices in smallholder pig farms in Madagascar. *Preventive veterinary medicine*, *92*(3), 199-209. doi: <https://doi.org/10.1016/j.prevetmed.2009.08.010>

Delsart, M., Pol, F., Dufour, B., Rose, N., & Fablet, C. (2020). Pig farming in alternative systems: strengths and challenges in terms of animal welfare, biosecurity, animal health and pork safety. *Agriculture*, *10*(7), 261. doi: <https://doi.org/10.3390/agriculture10070261>

Dewey, C., Bottoms, K., Carter, N., & Richardson, K. (2014). A qualitative study to identify potential biosecurity risks associated with feed delivery. *Journal of Swine Health and Production*, *22*(5), 232-243.

Filippitzi, M. E., Brinch Kruse, A., Postma, M., Sarrazin, S., Maes, D., Alban, L., ... & Dewulf, J. (2018). Review of transmission routes of 24 infectious diseases preventable by biosecurity measures and comparison of the implementation of these measures in pig herds in six European countries. *Transboundary and emerging diseases*, *65*(2), 381-398. doi: <https://doi.org/10.1111/tbed.12758>

Furutani, A., Sekiguchi, S., Sueyoshi, M., & Sasaki, Y. (2019). Effect of intervention practices to control the porcine epidemic diarrhea (PED) outbreak during the first epidemic year (2013–2014) on time to absence of clinical signs and the number of dead piglets per sow in Japan. Preventive veterinary medicine, 169, 104710. doi: <https://doi.org/10.1016/j.prevetmed.2019.104710>

Jurado, C., Martínez-Avilés, M., De La Torre, A., Štukelj, M., de Carvalho Ferreira, H. C., Cerioli, M., ... & Bellini, S. (2018). Relevant measures to prevent the spread of African swine fever in the European Union domestic pig sector. *Frontiers in veterinary science*, *5*, 77. doi: <https://doi.org/10.3389/fvets.2018.00077>

Kim, Y., Yang, M., Goyal, S. M., Cheeran, M. C., & Torremorell, M. (2017). Evaluation of biosecurity measures to prevent indirect transmission of porcine epidemic diarrhea virus. *BMC veterinary research*, *13*(1), 1-9. doi: <https://doi.org/10.1186/s12917-017-1017-4>

Kouam, M. K., Jacouba, M., & Moussala, J. O. (2020). Management and biosecurity practices on pig farms in the Western Highlands of Cameroon (Central Africa). *Veterinary Medicine and Science*, *6*(1), 82-91. doi: <https://doi.org/10.1002/vms3.211>

Kruse, A. B., Nielsen, L. R., & Alban, L. (2020). Herd typologies based on multivariate analysis of biosecurity, productivity, antimicrobial and vaccine use data from Danish sow herds. *Preventive veterinary medicine*, *181*, 104487. doi: <https://doi.org/10.1016/j.prevetmed.2018.06.008>

Kuster, K., Cousin, M. E., Jemmi, T., Schüpbach-Regula, G., & Magouras, I. (2015). Expert opinion on the perceived effectiveness and importance of on-farm biosecurity measures for cattle and swine farms in Switzerland. PLoS One, 10(12), e0144533. doi: <https://doi.org/10.1371/journal.pone.0144533>

Lambert, M. È., Poljak, Z., Arsenault, J., & D’Allaire, S. (2012). Epidemiological investigations in regard to porcine reproductive and respiratory syndrome (PRRS) in Quebec, Canada. Part 1: Biosecurity practices and their geographical distribution in two areas of different swine density. *Preventive Veterinary Medicine*, *104*(1-2), 74-83. doi: [https://doi.org/10.1016/j.prevetmed.2011.12.004](https://doi.org/10.1016/j.prevetmed.2011.12.004)

Léger, A., De Nardi, M., Simons, R., Adkin, A., Ru, G., Estrada-Peña, A., & Stärk, K. D. (2017). Assessment of biosecurity and control measures to prevent incursion and to limit spread of emerging transboundary animal diseases in Europe: An expert survey. *Vaccine*, *35*(44), 5956-5966. doi: <https://doi.org/10.1016/j.vaccine.2017.07.034>

Lewerin, S. S., Österberg, J., Alenius, S., Elvander, M., Fellström, C., Tråvén, M., ... & Jacobson, M. (2015). Risk assessment as a tool for improving external biosecurity at farm level. BMC Veterinary Research, 11(1), 1-10. doi: <https://doi.org/10.1186/s12917-015-0477-7>

Maes, D. G., Deluyker, H., Verdonck, M., De Kruif, A., Ducatelle, R., Castryck, F., ... & Vrijens, B. (2001b). Non‐infectious factors associated with macroscopic and microscopic lung lesions in slaughter pigs from farrow‐to‐finish herds. *Veterinary Record*, *148*(2), 41-46. doi: <https://doi.org/10.1136/vr.148.2.41>

Maes, D., Chiers, K., Haesebrouck, F., Laevens, H., Verdonck, M., & De Kruif, A. (2001a). Herd factors associated with the seroprevalences of Actinobacillus pleuropneumoniae serovars 2, 3 and 9 in slaughter pigs from farrow-to-finish pig herds. *Veterinary research*, *32*(5), 409-419. doi: <https://doi.org/10.1051/vetres:2001133>

Martinez, M., de la Torre, A., Sánchez-Vizcaíno, J. M., & Bellini, S. (2021). Biosecurity measures against African swine fever in domestic pigs. In *Understanding and combatting African Swine Fever: A European perspective* (pp. 45-53). Wageningen Academic Publishers. doi: <https://doi.org/10.3920/978-90-8686-910-7_10>

Niemi, J. K., Sahlström, L., Kyyrö, J., Lyytikäinen, T., & Sinisalo, A. (2016). Farm characteristics and perceptions regarding costs contribute to the adoption of biosecurity in Finnish pig and cattle farms. *Review of Agricultural, Food and Environmental Studies*, *97*(4), 215-223. doi: <https://doi.org/10.1007/s41130-016-0022-5>

Nöremark, M., & Sternberg-Lewerin, S. (2014). On-farm biosecurity as perceived by professionals visiting Swedish farms. *Acta Veterinaria Scandinavica*, *56*(1), 1-11. doi: <https://doi.org/10.1186/1751-0147-56-28>

Postma, M., Backhans, A., Collineau, L., Loesken, S., Sjölund, M., Belloc, C., ... & Dewulf, J. (2016a). Evaluation of the relationship between the biosecurity status, production parameters, herd characteristics and antimicrobial usage in farrow-to-finish pig production in four EU countries. *Porcine Health Management*, *2*(1), 1-11.doi: <https://doi.org/10.1186/s40813-016-0028-z>

Postma, M., Backhans, A., Collineau, L., Loesken, S., Sjölund, M., Belloc, C., ... & Dewulf, J. (2016b). The biosecurity status and its associations with production and management characteristics in farrow-to-finish pig herds. *Animal*, *10*(3), 478-489. doi: <https://doi.org/10.1017/S1751731115002487>

Raasch, S., Postma, M., Dewulf, J., & Stärk, K. D. C. (2018). Association between antimicrobial usage, biosecurity measures as well as farm performance in German farrow-to-finish farms. *Porcine health management*, *4*(1), 1-14. doi: <https://doi.org/10.1186/s40813-018-0106-5>

Sahlström, L., Virtanen, T., Kyyrö, J., & Lyytikäinen, T. (2014). Biosecurity on Finnish cattle, pig and sheep farms–results from a questionnaire. *Preventive Veterinary Medicine*, *117*(1), 59-67. doi: <https://doi.org/10.1016/j.prevetmed.2014.07.004>

Simon-Grifé, M., Martín-Valls, G. E., Vilar, M. J., García-Bocanegra, I., Martín, M., Mateu, E., & Casal, J. (2013). Biosecurity practices in Spanish pig herds: perceptions of farmers and veterinarians of the most important biosecurity measures. Preventive veterinary medicine, 110(2), 223-231. doi: <https://doi.org/10.1016/j.prevetmed.2012.11.028>

Stewart, S. C., Dritz, S. S., Woodworth, J. C., Paulk, C., & Jones, C. K. (2020). A review of strategies to impact swine feed biosecurity. *Animal Health Research Reviews*, *21*(1), 61-68. doi: <https://doi.org/10.1017/S146625231900015X>

Valeeva, N. I., Van Asseldonk, M. A. P. M., & Backus, G. B. C. (2011). Perceived risk and strategy efficacy as motivators of risk management strategy adoption to prevent animal diseases in pig farming. *Preventive veterinary medicine*, *102*(4), 284-295. doi: <https://doi.org/10.1016/j.prevetmed.2011.08.005>

Wu, Q., Schulz, L. L., Tonsor, G. T., & Smith, J. M. (2017). Expert views on effectiveness, feasibility, and implementation of biosecurity measures for mitigating tier 1 disease risks in the US swine, beef cattle, and dairy industries. *J. Vet. Sci. Technol*, *8*(435), 10-4172. doi: 10.4172/2157-7579.1000435
